# Evaluation of Glycerylphytate Crosslinked Semi- and Interpenetrated Polymer Membranes of Hyaluronic Acid and Chitosan for Tissue Engineering

**DOI:** 10.3390/polym12112661

**Published:** 2020-11-11

**Authors:** Ana Mora-Boza, Elena López-Ruiz, María Luisa López-Donaire, Gema Jiménez, María Rosa Aguilar, Juan Antonio Marchal, José Luis Pedraz, Blanca Vázquez-Lasa, Julio San Román, Patricia Gálvez-Martín

**Affiliations:** 1 Institute of Polymer Science and Technology, ICTP-CSIC, C/Juan de la Cierva 3, 28006 Madrid, Spain; amorboz@gmail.com (A.M.-B.); mraguilar@ictp.csic.es (M.R.A.); jsroman@ictp.csic.es (J.S.R.); 2 CIBER-BBN, Health Institute Carlos III, C/Monforte de Lemos 3-5, Pabellón 11, 28029 Madrid, Spain; joseluis.pedraz@ehu.eus; 3 Biopathology and Regenerative Medicine Institute (IBIMER), Centre for Biomedical Research, University of Granada, E-18100 Granada, Spain; elenalopru@gmail.com (E.L.-R.); gemajg@ugr.es (G.J.); jmarchal@go.ugr.es (J.A.M.); 4 Instituto de Investigación Biosanitaria de Granada (ibs.GRANADA), University Hospitals of Granada University of Granada, E-18071 Granada, Spain; 5 Department of Health Sciences, University of Jaén, 23071 Jaén, Spain; 6 Excellence Research Unit “Modeling Nature” (MNat), University of Granada, E-18016 Granada, Spain; 7 Department of Human Anatomy and Embryology, Faculty of Medicine, University of Granada, E-18016 Granada, Spain; 8 NanoBioCel Group, Laboratory of Pharmaceutics, University of the Basque Country (UPV/EHU), School of Pharmacy, Paseo de la Universidad 7, 01006 Vitoria-Gasteiz, Spain; 9 R&D Human Health, Bioibérica S.A.U., 08950 Barcelona, Spain; pgalvez@bioiberica.com

**Keywords:** interpenetrated polymer network, semi-IPN, methacrylated hyaluronic acid, chitosan, glycerylphytate, mesenchymal stem cell

## Abstract

In the present study, semi- and interpenetrated polymer network (IPN) systems based on hyaluronic acid (HA) and chitosan using ionic crosslinking of chitosan with a bioactive crosslinker, glycerylphytate (G_1_Phy), and UV irradiation of methacrylate were developed, characterized and evaluated as potential supports for tissue engineering. Semi- and IPN systems showed significant differences between them regarding composition, morphology, and mechanical properties after physicochemical characterization. Dual crosslinking process of IPN systems enhanced HA retention and mechanical properties, providing also flatter and denser surfaces in comparison to semi-IPN membranes. The biological performance was evaluated on primary human mesenchymal stem cells (hMSCs) and the systems revealed no cytotoxic effect. The excellent biocompatibility of the systems was demonstrated by large spreading areas of hMSCs on hydrogel membrane surfaces. Cell proliferation increased over time for all the systems, being significantly enhanced in the semi-IPN, which suggested that these polymeric membranes could be proposed as an effective promoter system of tissue repair. In this sense, the developed crosslinked biomimetic and biodegradable membranes can provide a stable and amenable environment for hMSCs support and growth with potential applications in the biomedical field.

## 1. Introduction

Hydrogels derived from natural polymers exhibit potential for tissue engineering (TE) applications as they closely mimic the extracellular matrix (ECM) of native tissues. They also provide a suitable environment for supporting cell adhesion and growth compared to other materials due to their biocompatibility, swelling ability, and possibility of diffusion for nutrients and waste exchange [1,2]. Thus, hydrogel membranes that present mechanical and physicochemical properties similar to those of native tissues have gained much attention in the latest years [3].

Polysaccharides-based hydrogels are promising candidates to fulfil the diversified demands in a variety of biomedical applications [4]. Chitosan (Ch) and hyaluronic acid (HA) are two polysaccharides that can form hydrogels and are widely exploited for its use as scaffolds for TE [2]. Ch is a linear polysaccharide widely applied in the biomedical field due to its structural similarity to the naturally occurring glycosaminoglycans and its susceptibility to degradation by enzymes in humans [2,5]. Ch shows also antimicrobial and hemostatic properties attributed to its cationic nature of amino groups [4,5,6]. HA is a glycosaminoglycan found in ECM that plays a key role as an environmental cue to regulate cell behavior during embryonic development, healing processes, inflammation [4,7,8]. HA participates in important cell signaling pathways due to the presence of cell surface receptors like CD44 and RHAMM, which is a receptor for hyaluronan-mediated motility [9]. Moreover, HA demonstrated to play powerful multifunctional activity in homeostasis and tissue remodeling processes [6,10]. Ch and HA have been combined to fabricate different matrices for several TE applications [1,2,4,11,12,13,14,15,16,17], such as polyelectrolyte complexes for dental pulp regeneration [12] or injectable hydrogels [1,2,14,17,18] for cartilage repair [14,18], peripheral nerve regeneration [17], and adipose tissue regeneration [19], among others. Ch and HA combination has been particularly attractive for osteochondral regeneration applications due to their physicochemical and compositional similarities with native cartilage [7,11,20,21,22]. For example, Mohan et al. [11] performed a profound study about the regeneration capacity of Ch/HA gels on critical osteochondral defects in knee joints of New Zealand white rabbits, claiming the potential regenerative capacities of their systems. In other work carried out by Erickson et al. [7], HA and Ch were used to fabricate a bilayer scaffold to repair osteochondral defects, showing excellent cellular proliferation results.

Among all available types of polymeric-based matrices, interpenetrating networks (IPNs) and semi-IPNs membranes provide highly tunable platforms regarding composition and physicochemical properties by the combination of different polymers and crosslinking processes. These systems have showed attractive features in terms of enhanced stability and mechanical properties, mainly due to the molecular reinforcement resulted from the network/s of different polymers [4,9,23]. As it is known, an IPN consists of a combination of two (or more) polymer networks which are physically or chemically crosslinked and entangled within each other. For its part, in a semi-IPN, only one of the polymers is crosslinked and the linear polymer is entangled within the network [9]. The present approach provides a promising candidate system for TE applications in the form of natural-occurring polysaccharides semi- and IPN systems using a novel recently developed crosslinker glycerylphytate (G_1_Phy). G_1_Phy is a natural derived crosslinker that possesses reduced cytotoxicity and antioxidant properties [24], and showed enhanced cellular adhesion and proliferation in comparison to other traditionally used phosphate-based crosslinkers like tripolyphosphate [25]. Specifically, we develop and evaluate semi- and IPN systems formed by Ch/HA and Ch/methacrylated HA (HAMA), respectively, ionically crosslinked with G_1_Phy [25]. Although Ch [4] and HA [9,20,26,27] have been combined with other polymers for the preparation of semi- and IPN systems, the reported polymeric composition and applied crosslinking strategies in this work has not been explored before. The obtained materials were characterized by a set of techniques in terms of composition, physicochemical, morphological and mechanical properties as well as in vitro behavior, observing clear differences that are expected to influence its efficacy on their biological performance. Biological assays regarding viability, cell adhesion and proliferation were assessed on human mesenchymal stromal cells (hMSCs). The excellent biocompatibility of our systems was demonstrated by large spreading areas of hMSCs on the hydrogel surfaces. Moreover, semi-IPN system showed a significantly enhancement of hMSCs proliferation over time in comparison to the other systems. Our findings suggested that surface properties and composition, can play a key role in the final application of semi- and IPN membranes as effective matrices for TE, tentatively to guided bone regeneration applications.

## 2. Materials and Methods

### 2.1. Materials

HA (Ophthalmic grade, 800–1000 kDa, Bioiberica, Barcelona, Spain) and Ch with a degree of deacetylation of 90% (Medical grade, M_w_: 200–500 kDa, Altakitin SA, Lisboa, Portugal) were used as received. Methacrylic anhydride (MA), poly (ethylene glycol) dimethacrylate (PEGDMA, M_n_: 8000 Da) and the photoinitiator Irgacure 2959 were purchased from Sigma Aldrich (St. Louis, MO, USA) and used as received. G_1_Phy was prepared as previously described by Ana Mora-Boza et al. [24], using phytic acid and glycerol from Sigma Aldrich. Solvents as isopropanol (Scharlau, Barcelona, Spain) and ethanol (BDH Chemicals, Philadelphia, PA, USA) were used as received. Dialysis membranes (3500 Da cut off) were purchased from Spectrum^®^ (Columbia, MO, USA). Additional reagents such as phosphate buffered saline (PBS), calcium chloride, nitric acid 65% (v/v), acetic acid (AA) and sodium hydroxide were purchased from Thermo Fisher Scientific Corporation (Waltham, MA, USA). Tris hydrochloride, 1 M solution (pH 7.5/Mol. Biol.) was purchase from Fisher BioReagents (Waltham, MA, USA).

### 2.2. Synthesis of Methacrylated Hyaluronic Acid

HAMA was synthesized through an esterification reaction in alkaline conditions following the protocol described by Khunmaneeet et al. [28]. HA (1 g) was dissolved in 100 mL of Milli-Q water in a two necked glass flask for 24 h. MA was added to the HA solution at a MA:HA ratio of 1:1. The mixture was kept at 0 °C using an ice bath and the pH was controlled at 8.5 by adding NaOH (5 M) with the help of an automatic titrator (Metrohm, Switzerland) for 24 h. The final product was purified by precipitation in cold ethanol, subsequently centrifuged (Eppendorf centrifuge 5810 R model, Madrid, Spain), dissolved in double distilled water (ddH_2_O), and dialyzed for 4 days. After freeze drying, a white powder was finally obtained. HAMA was characterized by proton nuclear magnetic resonance (^1^H-NMR, Bruker AVANCE IIIHD-400, MA, USA) and attenuated total reflection–Fourier transform infra-red (ATR-FTIR, Perkin-Elmer (Spectrum One), Waltham, MA, USA) spectroscopies. HAMA methacrylation degree was determined by its ^1^H-NMR spectrum giving a value of 4.5% (Figure S1).

### 2.3. Preparation of Ch Membranes

Dried Ch was dissolved at a concentration of 2 wt.% in 1% AA water solution containing 13 wt.% CaCl_2_ respect to Ch. Once it was dissolved, it was poured into a glass petri dish (internal diameter: 49 mm) and dried under moister conditions at room temperature until constant weight. Then, membranes were detached from the petri dishes after 5 min of incubation in NaOH and subsequently rinsed with Milli-Q water until neutral pH was reached. Finally, membranes were ionically crosslinked by their immersion into a G_1_Phy water solution at a concentration of 15 mg/mL (30 wt.% respect to chitosan) for 24 h and room temperature. The uncoupled G_1_Phy was removed by rinsing twice the membranes with Milli-Q water.

### 2.4. Preparation of Ch/HA and Ch/HAMA Membranes

**Figure 1:**
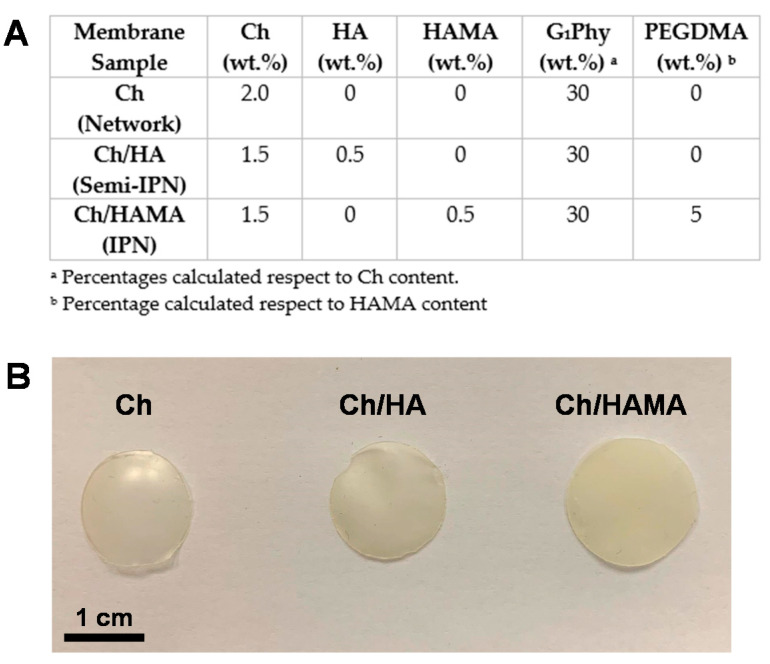
Polymer and crosslinker concentrations (wt.%) used for the fabrication of Ch membranes, semi-, and IPNs developed in this work (**A**); Digital images of the developed systems (**B**).

Either type of membranes, Ch/HA or Ch/HAMA, were prepared with a content of 75% of Ch and 25% of HA or HAMA, respectively. Either HA or HAMA solution in 1% AA with CaCl_2_ (%) was added to the Ch solution together with additional drops of 2 M HCl to achieve the total dissolution of both polymers. For Ch/HAMA membranes, HAMA solution was supplemented with 5% PEGDMA crosslinker and 2% of photoinitiator Irgacure 2959, both respect to HAMA content, in order to trigger photopolymerization by UV-light irradiation. Therefore, the corresponding solution was poured and irradiated at 365 nm for 15 min using a UVP chamber photoreactor (CL-1000, Thermo Fisher Scientific Corporation, MA, US), equipped with 5 bulbs of 365 nm working at an intensity of 2.9 mW/cm^2^. Finally, membranes were submitted to ionic crosslinking with G_1_Phy following the same protocol as described in Section 2.3. All membranes were prepared in the form of a circle of 5 cm diameter using a petri dish and with thicknesses between 0.22 ± 0.03 to 0.42 ± 0.06 mm. Those membranes were punched with diameters of 12 mm for the in vitro experiments and biological assays. Figure 1 shows the polymer and crosslinker compositions used for the fabrication of each system along with the digital images of the as-obtained membranes.

### 2.5. Characterisation Techniques

^1^H-NMR spectra were recorded with a Varian Mercury 400 MHz (Agilent, Santa Clara, CA, USA). The spectra were carried out at 25 °C in D_2_O (10% w/v) and referenced to the residual proton absorption of the solvent, D_2_O [4.7 ppm].

ATR-FTIR of samples were carried out on a Perkin-Elmer Spectrum BX spectrophotometer (MA, USA). All spectra were recorded from 600 to 4000 cm^−1^ with a resolution of 4 cm^−1^ and 32 scans.

Elemental analysis (EA) was performed with an elemental LECO model CHNS-932 microanalyzer (MI, USA). The determination of C and H was carried out with CO_2_ and H_2_O specific infrared detectors, while N (N_2_) was determined by thermic conductivity. The measurements were conducted at 990 °C using He as transporter gas.

Inductively coupled plasma optical emission spectrometry (ICP-OES) measurements were carried out in a 4300 DV Perkin-Elmer plasma emission spectrometer (MA, USA) under dynamic argon flow at 16 L/min using a Gemcone (Perkin-Elmer, MA, USA) nebulizer under dynamic argon flow at 0.8 L/min, and 1300 W of plasma power.

Scanning electron microscopy (SEM) images were taken in a Hitachi S-8000 instrument (Tokyo, Japan) operating in transmission mode at 100 kV on dry samples.

Atomic force microscopy (AFM) analysis was performed with an apparatus PicoLE (Molecular Imaging) operating in the acoustically driven, intermittent contact (“tapping”) mode, using standard silicon AFM probes (NSC11/Cr-Au, Mikromasch, Tallinn, Estonia) having a cantilever spring constant of 48 N/m and a resonance frequency of 330 kHz. 10 × 10 mm^2^ AFM images were taken on dry samples. Topography was examined by AFM using the WSxM 5.0 Develop 9.1 software. Three acquisitions were made with roughness parameters analysis for each sample. Data were expressed as mean ± standard deviation (SD).

Water contact angle (WCA) measurements were performed at 25 °C on dried membranes, by the sessile drop technique using a KSV instruments LTD CAM 200 Tensiometer (Hertfordshire, UK) and employing Milli-Q water as a liquid with known surface tension. A minimum of 10 measurements were taken and averaged for each sample. Data were expressed as mean ± SD.

Rheological measurements were determined using an advanced rheometer from TA instruments, model AR-G2 (DE, US), equipped with a Peltier and a solvent trap. The last one allows leading the measurement in a water-saturated atmosphere by avoiding water evaporation from the membrane. Samples were previously stabilized by their immersion for 24 h in 7.4 PBS at 37 °C. All tests were carried out using a 25 mm diameter steel sand blasted parallel plate. Oscillatory shear tests with strain sweep step were performed at a frequency of 0.5 Hz and a strain ranging from 0.01 to 100% in order to determine the linear viscoelastic region (LVR) of the different membranes. Finally, frequency sweeping tests of membranes were conducted with a frequency scanning from 0.01 to 10 Hz at 0.1% strain and 37 °C to determine the elastic (G’) and viscous (G”) moduli. Three replicates of each sample were evaluated.

The average mesh size *ξ* was calculated from *G*’ based on the rubber elasticity theory (RET) using the following Equation (1) [29]:

(1) $$\xi ={\left(\frac{G\prime N_{A}}{RT}\right)}^{-\frac{1}{3}}$$

where *G*’ is the storage modulus, *N_A_* is the Avogadro constant, *R* is the molar gas constant, and *T* is the temperature. Three replicates of each sample were evaluated. Data were expressed as mean ± SD.

### 2.6. G_1_Phy Quantification

The amount of G_1_Phy ionically crosslinked in the membranes was quantified measuring the P content using ICP-OES (Perkin-Elmer, MA, US). Polymeric membranes were dried at 60 °C after their incubation in G_1_Phy solution until constant weight. Afterwards, membranes were digested in nitric acid, 65% (v/v) at 60 °C for 24 h. Then, samples were diluted with Milli-Q water to 5% nitric acid concentration in order to be compatible for ICP-OES analysis. A blank solution was prepared with only nitric acid at 5% in Milli-Q water. All measurements were performed in triplicate. A standard calibration curve of P with concentration from 0–1000 mg/L was used. Three measurements were made for each sample. Data were expressed as mean ± SD.

### 2.7. Swelling

Dry membranes with a diameter of 12 mm were soaked in 10 mL of PBS (pH = 7.4) at 37 °C. The swollen membranes were removed at different periods of time. After removing the attached excess of water on the surface with filter paper, the membranes were weighed. The water uptake was calculated as described in Equation (2):

Swelling (%) = [(W_W_ − W_0_)/W_0_] × 100 (2)

where W_w_ and W_0_ are the weight of the swollen membrane at time t, and the initial dried weight of the membrane, respectively. For each period of time and sample, a minimum of four replicates were measured and averaged. Data were expressed as mean ± SD.

### 2.8. Degradation

Dry membranes (12 mm diameter and 0.2 mm thickness) were weighed and then placed in 10 mL of PBS (pH 7.4) at 37 °C. The membranes were retrieved at predetermined time point and washed with Milli-Q water to remove any remaining salts. The membranes were then dried at 60 °C until constant weight. The percentage of weight loss was calculated following the Equation (3):

Weight loss (%) = [(W_W_ − W_t_)/W_0_] × 100 (3)

where W_0_ and W_t_ are the weight of the initial dry membrane and the dried membrane at time t after incubation in PBS, respectively. For each period of time and sample, a minimum of four replicates were measured and averaged. Data were expressed as mean ± SD.

### 2.9. Release of G_1_Phy

The corresponding membrane (12 mm diameter) was immersed into 10 mL of Tris-HCl 0.1 M buffer (pH 7.4) and incubated at 37 °C. Aliquots of 2 mL were taken at different periods of time (1,2,4,7 and 14 days) and replaced with fresh media. The different aliquots were diluted to 5 mL with Milli-Q water and measured by ICP-OES. For each period of time and sample, a minimum of four replicates were measured and averaged. Data were expressed as mean ± SD.

### 2.10. Cell Studies

#### 2.10.1. hMSCs Isolation and Culture from Adipose Tissue

hMSCs used in this study were isolated from human abdominal fat obtained from healthy donors undergoing liposuction plastic surgery. Ethical approval for the study was obtained from the Ethics Committee (number: 02/022010) of the Clinical University Hospital of Málaga, Spain. Informed patient consent was obtained for all samples used in this study. hMSCs were isolated from human adipose tissue and characterized as previously reported [30,31]. Cells were cultured in high-glucose Dulbecco’s modified Eagle’s medium (DMEM; Sigma-Aldrich, St Louis, MO, USA) supplemented with 10% fetal bovine serum (FBS; Sigma-Aldrich), 100 U/mL penicillin and 100 mg/mL streptomycin (Invitrogen Inc., Grand Island, NY, USA) at 37 °C in a humidified atmosphere containing 5% CO_2_. Medium was regularly changed every 3 days. At 80% of confluence, cells were subcultured. For all the experiments cells were used between passages 4 and 6.

#### 2.10.2. hMSCs Culture in Hydrogel Membranes

hMSCs isolation and culture from adipose tissue were performed as a described in Section 2.10.1. Hydrogel membranes (12 mm diameter) were sterilized by immersion in 70% ethanol aqueous solution for 1 h, washed several times in PBS and then subjected to UV light (Philips, Pila, Poland) for 20 min on both sizes. Then, hydrogel membranes were incubated in 24 well plates with complete medium overnight before cells were seeded. hMSCs suspension containing 30,000 cells in 200 µL of medium was slowly dropped onto the surface of each membrane and incubated for 2 h at 37 °C. After that, 1 mL of fresh medium was added to each well plate. All samples were incubated under a 5% CO_2_ atmosphere at 37 °C. The culture medium was replaced every 2 days and the hydrogel membranes were processed for subsequent analysis.

#### 2.10.3. Cell Viability Assay

Cell viability was determined on days 1, 7 and 21 using Live/Dead™ Viability/Cytotoxicity Kit (Invitrogen Inc., Grand Island, NY, USA). The hydrogel membranes were incubated in PBS containing Calcein AM (2 µM) and ethidium homodimer (4 µM) at 37 °C for 30 min to stain live and dead cells, respectively. Membranes were imaged by confocal microscopy (Nikon Eclipse Ti-E A1, Amsterdam, The Netherlands) and analyzed using NIS-Elements software (Amsterdam, The Netherlands).

#### 2.10.4. Cell Proliferation Assay

Cell proliferation was analyzed using AlamarBlue^®^ assay (Bio-Rad Laboratories, Inc., manufactured by Trek Diagnostic System., Hercules, CA, USA) after 1, 5, 7, 14 and 21 days. The hydrogel membranes were incubated with AlamarBlue^®^ solution at 37 °C for 3 h. Fluorescence of reduced AlamarBlue^®^ was determined at 530/590 nm excitation/emission wavelengths (Synergy HT, BIO-TEK, Winooski, VT, USA).

#### 2.10.5. Environmental Scanning Electron Microscopy (ESEM)

The hydrogel membranes were analyzed using a variable-pressure equipment FEI, mod. Quanta 400 (OR, USA). The analysis was performed to characterize the surface structure of the membranes and cell growth after 21 days in culture. Samples were fixed with 2% glutaraldehyde and, then, were rinsed in 0.1 M cacodylate buffer and incubated overnight at 4 °C. For critical point the samples were then maintained with osmium tetroxide 1% at room temperature during 1 h and dehydrated in a series of ethanol solutions (50%, 70%, 90% and 100%) by soaking the samples in each solution for 15 min. Subsequently, samples were critical point dried (Anderson, 1951) in a desiccator (Leica EMCPD300, Wetzlar, Germany) and covered by evaporating them in a carbon evaporator (Emitech K975X).

#### 2.10.6. Statistical Analysis

All graphed data represent the mean ± SD from at least three experiments. Two-tailed Student T test analysis were performed for Ch/HA and Ch/HAMA samples with respect to Ch ones at each time point at significance level of ** *p* < 0.01, and for Ch/HA samples with respect to Ch/HAMA samples at each time point at significance level of (## *p* < 0.01).

## 3. Results and Discussion

### 3.1. Physicochemical Characterization and Viscoelastic Properties of Membranes

Elemental composition of the membranes was determined by elemental analysis. Table 1 shows the theoretical and experimental elemental compositions (C, H, and N) for the different membranes. Experimental compositions correlated very well with those calculated theoretically, which revealed the absence of impurities. Table 1 also shows a decrease of the experimental C/N value (5.25 ± 0.07) respect to the theoretical one (6.1) for Ch/HA membranes. In case of Ch/HAMA membranes, the experimental C/N ratio (5.83 ± 0.15) approached the theoretically expected (6.1). The decrease of C/N ratio in semi-IPN systems could indicate a possible release of HA from these membranes after washing steps since this phenomenon was not observed in IPN systems. This means that the covalent crosslinking of HAMA mediated by UV-light seemed to retain the HA polysaccharide in the IPN. In addition, this fact was confirmed by a decrease in the yield percentage of Ch/HA membranes. Nevertheless, both semi- and IPN systems can be considered promising candidates for tissue regeneration since both of them contain HA, an essential component of the native ECM [7]. Likewise, the presence of HA in the membranes will help to maintain the functionality and characteristic structure of regenerated tissues, which is essential for the final success of these scaffolds [6,11]. It is important to consider that the concentration range in which HA can provide these beneficial properties is quite wide [32].

The content of G_1_Phy incorporated in the membranes resulted from the ionic crosslinking between amino and phosphate groups was analyzed by ICP-OES (Table 1). The amount of the phytate crosslinker for Ch/HA membrane decreased somewhat compared to that of Ch sample what was attributed to the lower chitosan content in the former membranes (Ch:HA ratio = 75:25). However, the G_1_Phy crosslinked in the Ch/HAMA membranes decreased nearly to the half. This less crosslinker content in the IPN membranes in comparison to semi-IPNs maybe due to the fact that the covalently crosslinked HA could hindrance the availability of amine groups of chitosan for ionic interactions with the phosphate groups of G_1_Phy.

The FTIR spectra of the membranes are represented in Figure S2. The FTIR spectra of semi- and IPN samples showed the characteristic bands of Ch and HA polysaccharides. The main bands appeared between 3600 and 3200 cm^−1^ (ʋ O-H and N–H associated); at 2924 and 2854 cm^−1^ (ʋ C–H); at 1720 cm^−1^ (ʋ C = O in carboxylic and ester groups); at 1643/1634 cm^−1^ (ʋ C = O of amide, amide I); at 1579 cm^−1^ (δ N–H); at 1420 cm^−1^ (ʋ COO^-^ and δ C–H); at 1373 cm^−1^ (δ –CH_3_ symmetrical); at 1333 cm^−1^ (ʋ C–N, amide III); at 1258/1262 cm^−1^ (X P = O); at 1150 cm^−1^ (υ C–O–C asymmetric); at 1066, 1029, 995 and 984 cm^−1^ (ʋ C–O alcohols, ʋ P–O and P–O–C, ʋ C–O glycosidic linkages and vibration of pyranose structure); at 893 and 721 cm^−1^ (υ P–O and P–O–C) [33,34].

**Figure 2:**
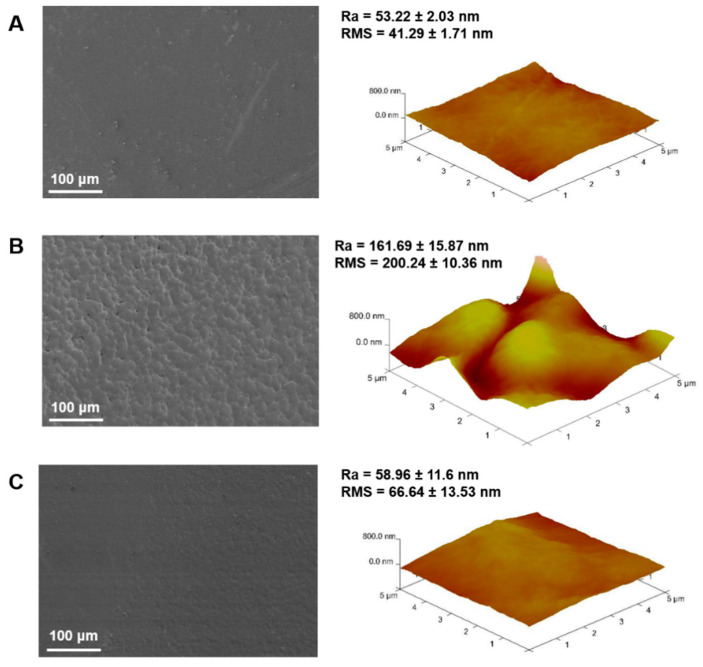
SEM micrographs (left) and AFM 3D perspective images with their respective calculated roughness parameters (right) for Ch (**A**), Ch/HA (**B**), and Ch/HAMA (**C**) polymeric membranes.

Surface morphology is a critical factor for the development of biomaterials that effectively promote cell adhesion and proliferation [35]. Figure 2 shows a detailed examination of surface topography for Ch (A), Ch/HA (B) and Ch/HAMA (C) by SEM and AFM, as well as the calculated roughness parameters (R_a_, roughness average, and RMS, root mean square) for the different systems. Qualitative topographic differences among the membranes can be observed in the SEM micrographs. Particularly, Ch system showed the flattest surface followed by the semi-IPN sample, while a much more granular surface was observed in semi-IPN system. This result illustrated a topographic change due to the incorporation of HA that can be a consequence of electrostatic interactions between carboxylic groups of HA and amino groups of Ch, which leads to polyelectrolyte complex formation [32]. The Ch-HA interactions could be hindered in IPN systems by UV curing process, resulting in flatter surfaces as it is observed in their Ch/HAMA micrographs. AFM 3D images show the nano features and the roughness parameters of representative areas of the membranes. R_a_ and RMS values correlated very well with the topography observed by SEM. As it was expected, R_a_ and RMS values were significantly higher for Ch/HA membranes in comparison to those of Ch and Ch/HAMA that were very similar to each other. Therefore, we can conclude that covalent crosslinking of HAMA with UV-light resulted in a more compact framework in IPN systems compared to semi-IPNs, which leaded to a decrease of roughness at the nanoscale [9].

Surface parameters such as wettability are important properties that must be studied since hydrophilic-hydrophobic balance greatly determines cell adhesion and proliferation properties of the scaffolds [36]. Surface-wetting characterization is currently carried out by WCA measurements the most common method being the sessile drop goniometry [37], which was used in this work. Measured WCA values for Ch, Ch/HA, Ch/HAMA membranes were 48.18 ± 2.71°, 40.97 ± 3.26°, and 47.73 ± 4.96°, respectively. All the systems showed hydrophilic surfaces (WCA < 90°) as it was expected because of the characteristic water absorption nature of these polysaccharides [38]. Different WCA for Ch samples are reported in literature. For instance, Tamer et al. found higher WCA values (89 ± 0.6°) for Ch surfaces [39] than those obtained in our work. However, it has been reported that Ch polarity highly depends on the type and concentration of the used neutralization solution as well as the time of washing steps. Noriega et al. [40] performed a profound study where they reported a wide range of WCA for Ch surfaces, from 45 to 65°, that highly depended on neutralization parameters. As expected, hydrophilicity increased as neutralization base concentration and incubation time increased [40]. In our samples, the relatively low WCA values observed for Ch could also be due to the contribution of available phosphate groups coming from the G_1_Phy crosslinker on the membrane surface, which exhibits high affinity to polar liquids [41]. A decrease of WCA was observed for semi-IPN due to the higher content of G_1_Phy in this sample, along with the presence of HA and its polyanionic character [42]. For its part, IPNs showed WCA values similar to those of Ch membranes, which could be due to the reduction of carboxylic groups of HA after methacrylation reaction and further covalent crosslinking. Since membrane surfaces showed different WCA values, we can conclude that wettability properties seem to be an easily tunable parameter in function of composition and applied crosslinking processes in our systems.

**Figure 3:**
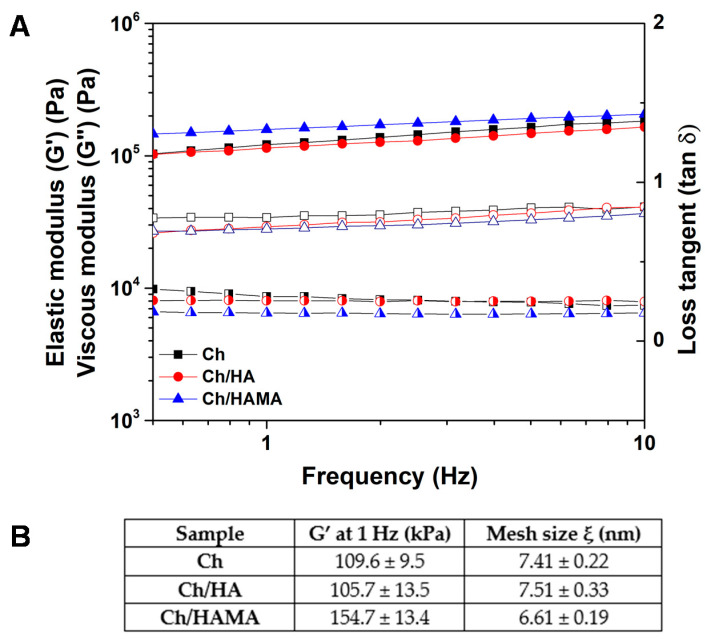
Evolution of elastic (G’, filled) and viscous (G’’, unfilled) moduli, and loss tangent (tan δ, half-filled) as a function of applied frequency at constant strain of 0.1% of Ch, Ch/HA, and Ch/HAMA polymeric membranes (**A**). The average mesh size values ξ of Ch, Ch/HA and Ch/HAMA polymeric membranes determined at a frequency of 1 Hz (**B**).

Rheological measurements were carried out to study the viscoelastic properties of our systems. The evolution of the elastic and viscous moduli of the membranes was studied in their LVR at a constant strain of 0.1%, and it is represented in Figure 3A. All the systems exhibited a plateau in the studied frequency range, which indicated the stability of the crosslinked network. This plateau also showed a solid-like behavior of the membranes, since elastic moduli was independent on the applied frequency [43]. IPN showed higher G’ values in comparison to Ch and semi-IPN systems due to the macromolecular reinforcement of the polymeric network after covalent crosslinking. IPNs have previously demonstrated to improve mechanical properties regarding semi-IPNs, because of double crosslinking mechanisms [4,9]. Finally, loss tangent (tan δ = viscous modulus/elastic modulus), which is an index of the viscoelasticity of the systems, was calculated by taking the ratio between G’’ and G’ at a frequency of 1 Hz. Values of 0.27, 0.17 and 0.24, were obtained for Ch, Ch/HA and Ch/HAMA membranes, respectively.

The mesh size is defined as the distance between crosslinking points of the membrane, which is related to the mechanical strength of the hydrogel [29,44]. As expected, the IPN membrane exhibited a lower mesh size value due to dual crosslinking, resulting in a more compact structure in comparison to Ch and semi-IPN membranes, which showed similar mesh size values. (Figure 3B) These results corroborate surface and morphology characterization illustrated in SEM and AFM images (Figure 2).

Collectively, the results described in this subsection showed some differences regarding composition, surface topography, wettability and mechanical properties for the semi- and IPN systems that are expected to also exert relevant differences on their swelling and degradation properties, as well as biological performance.

### 3.2. In Vitro Swelling and Degradation Studies

**Figure 4:**
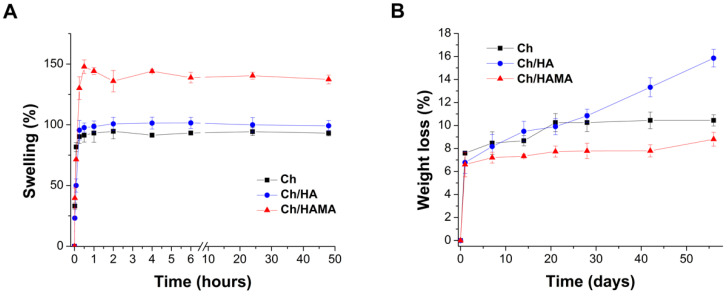
Effect of hydrogel membrane composition on swelling after incubation in PBS 7.4 at 37 °C for different periods of time (**A**). Weight loss of Ch, Ch/HA and Ch/HAMA membranes at different time points after soaking in PBS 7.4 at 37 °C under static conditions (**B**). Data represented the mean ± SD.

Swelling of the developed membranes was studied under physiological conditions and the results are represented in Figure 4A. For all membranes, a fast water uptake during the first hour was observed, reaching a stable value after 3 h, when equilibrium was attained. Ch and Ch/HA membranes showed rather similar swelling profiles giving final swelling values ~100% (Figure 4B). Due to pKa value of Ch at 6.4, its amino groups are not positively charged under physiological conditions and the repulsive forces in the polymeric backbone are not induced, not taking place this increase of the network water uptake [36]. For its part, Ch/HAMA membranes showed the highest equilibrium water absorption (up to 140%) which could be attributed to the formation of a dual crosslinked network able to locate a higher amount of water molecules in their interstices, and to retain a higher HA content in comparison to semi-IPN (Table 1). Nevertheless, all membranes showed moderate swelling that will contribute to maintain their structural stability. If necessary, swelling could be adjusted by varying the content of HAMA in the membrane [45].

In vitro degradation of all hydrogel membranes immersed in a PBS solution at 37 °C was below 10% over 14 days (Figure 4B) and it slightly increased after 28 days. However, for longer incubation time (~2 months), degradation of Ch and Ch/HAMA was maintained while Ch/HA membranes suffered further degradation (~16%). The initial membrane weight loss could be attributed to the progressive breaking of the ionic bonds formed between the phosphate and amino groups, what produces release of the G_1_Phy crosslinker and consequently, dissolution of HA and Ch polymeric chains. Similar results were reported for Ch/HA tissue engineering porous scaffolds where it was suggested than the degradation in PBS is only because of polymeric dissolution [46]. The higher weight loss of Ch/HA versus Ch membranes could be associated to the presence of entangled HA within the Ch network, favoring its dissolution. Accordingly, Ch/HAMA membranes, displayed the highest stability. This fact could be explained because the covalently crosslinked HA prevents its dissolution and the hydrolytically degradable ester bonds in the HAMA are sterically hindered [47].

### 3.3. G_1_Phy Release

**Figure 5:**
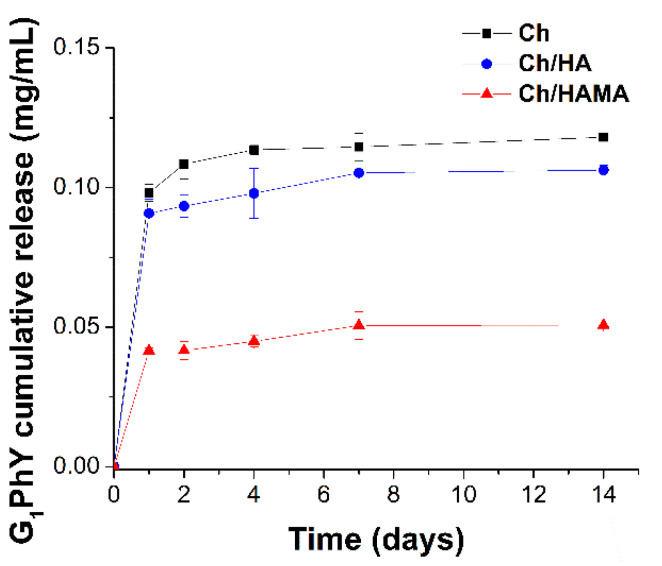
Release profiles of G_1_Phy from the Ch, Ch/HA and Ch/HAMA membranes in 0.1 M Tris buffer (pH 7.4) at 37 °C. Data represented the mean ± SD.

The release profile of G_1_Phy from the different hydrogel membranes is showed in Figure 5. All membranes showed a fast release (~72%) during the first 24 h which correspond to a G_1_Phy concentration of 0.1 ± 0.003, 0.09 ± 0.005 and 0.04 ± 0.001 mg/mL for Ch, Ch/HA and Ch/HAMA membranes, respectively. It is worth mentioning that at 24 h the G_1_Phy concentration of Ch/HAMA membrane is nearly half to that observed for Ch and Ch/HA membranes and this fact is in agreement with the lower initial content of G_1_Phy incorporated in the Ch/HAMA membranes, data previously described in Table 1. A plateau in the release profile is observed for the three systems after 7 days, where Ch and Ch/HA membranes reached an 85% release of the initial G_1_Phy membrane content, giving a final G_1_Phy concentration of 0.11 ± 0.004 and 0.10 ± 0.0005 mg/mL, respectively. On the other hand, Ch/HAMA membranes showed a G_1_Phy release of ~90%, corresponding to a final concentration of 0.05 ± 0.005 mg/mL. The slightly higher G_1_Phy release in the latter membrane could be due to the higher water uptake of Ch/HAMA membranes what can favor ion diffusion and subsequently ion exchange between G_1_Phy anions and negative anions present in the Tris buffer. Electrostatic interaction between G_1_Phy ions (PO_4_^2−^ or HPO_4_^−^) and the amino groups of Ch and breaking of links with incubation time could account for these release profiles. However, no complete release of initial G_1_Phy content was achieved after 14 days. Physical mixture of Ch with phytic acid has been reported by Barahuie et al. [48] showing a complete release after 60 s. Finally, it is worth mentioning that the release pattern of our systems is in agreement with that of Ch/phytic acid systems reported in the literature [48,49].

### 3.4. Biological Evaluation

#### 3.4.1. ESEM Microscopy of Hydrogel Membranes

**Figure 6:**
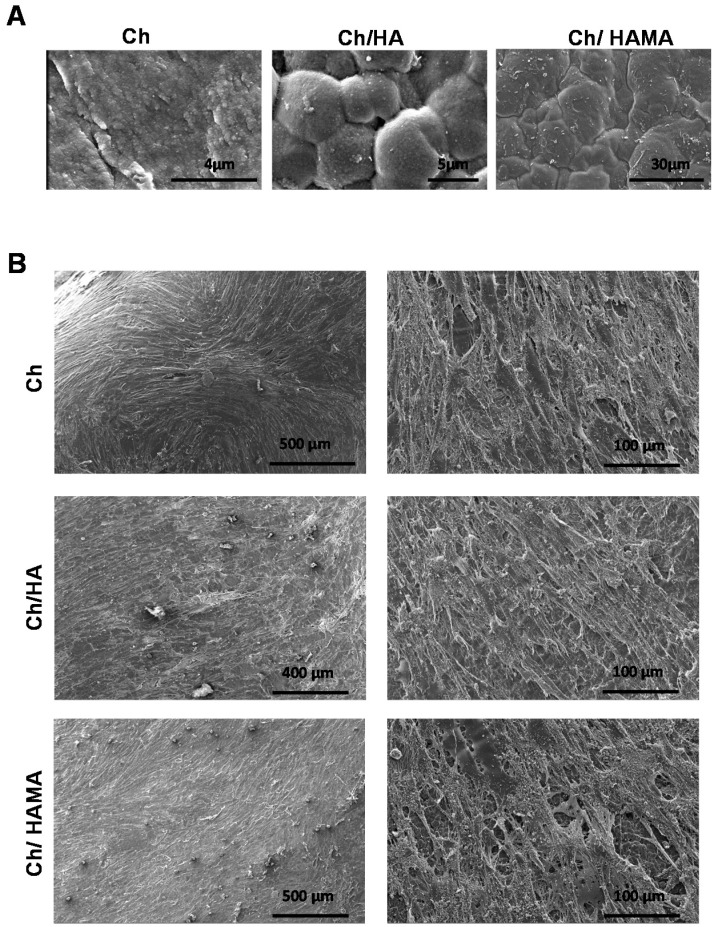
ESEM images of Ch, Ch/HA and Ch/HAMA hydrogel membranes. Representative ESEM images of hydrogel membranes before hMSCs culture (**A**). Representative ESEM images of hMSCs growing on hydrogel membranes after 21 days (**B**).

To characterize the microstructural architecture of Ch, Ch/HA and Ch/HAMA membranes and observe the morphology of hMSCs cultured on them, an ESEM analysis was carried out on day 21. It is known that the surface roughness is an important factor in promoting cell attachment [50]. ESEM images of Ch, Ch/HA and Ch/HAMA membranes (Figure 6A) revealed a rougher surface for the Ch/HA membrane compared to those of Ch/HAMA and Ch, corroborating the SEM observations for dried samples (Figure 2) but surfaces of all membranes were able to support cell growth (Figure 6B). ESEM images evidenced the cells covering the surface of Ch, Ch/HA and Ch/HAMA membranes, with good adhesion, spreading, and a homogenous distribution throughout the entire surface. Moreover, ESEM images showed an interconnected cell community that attached to the scaffold which also confirmed the biocompatibility of the membranes [51,52].

#### 3.4.2. Cell Viability and Proliferation Assays

**Figure 7:**
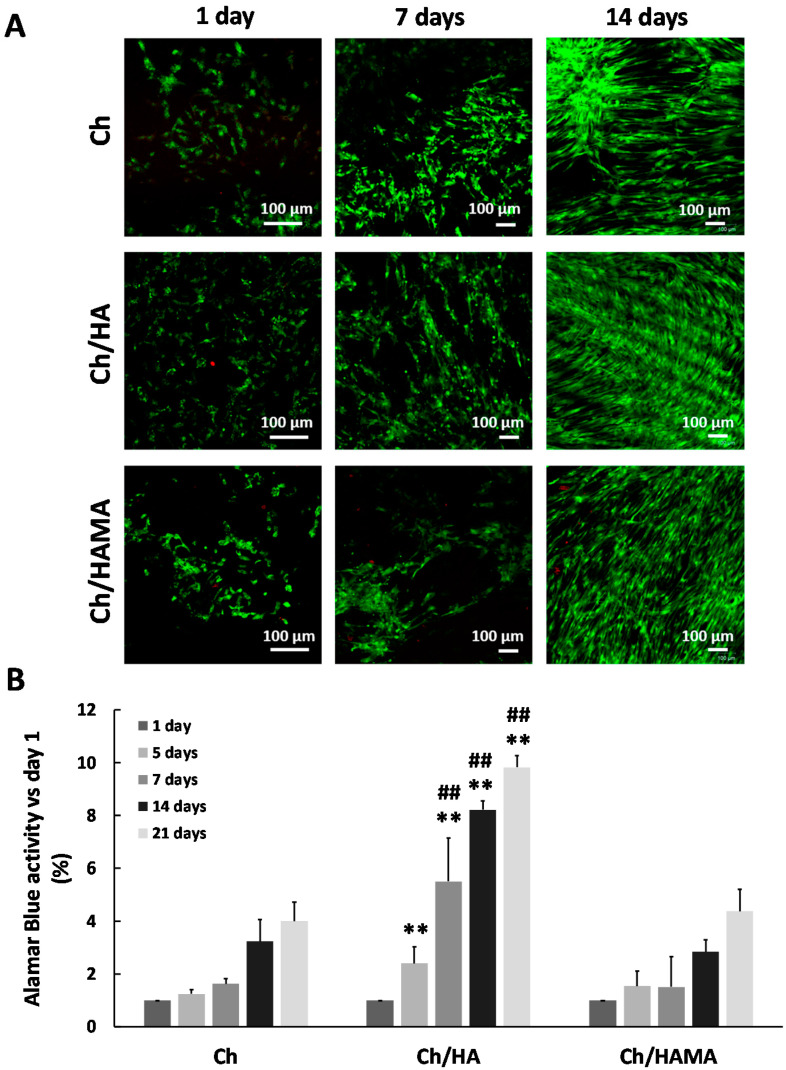
Cytocompatibility of Ch, Ch/HA and Ch/HAMA hydrogel membranes with hMSCs. Representative confocal images of hMSCs stained with Calcein AM (living cells in green) and ethidium homodimer (dead cell in red) at days 1, 7 and 21 using the Live/Dead^®®^ assay (**A**). Cell proliferation at the hydrogel membranes after 1, 5, 7, 14 and 21 days (**B**). Values are represented as mean ± SD (n = 3) and normalized respect to day 1 values. Two-tailed Student *T* test analysis were performed for Ch/HA and Ch/HAMA samples with respect to Ch samples at each time at significance level of ** *p* < 0.01, and for Ch/HA samples with respect to Ch/HAMA samples at each time point at significance level of (## *p* < 0.01).

In order to evaluate the feasibility of Ch, Ch/HA and Ch/HAMA hydrogel membranes as an adequate support for cell survival, the viability of the seeded hMSCs on the top of the membranes was evaluated. The live/dead assay was employed to visualize the presence of living and dead cells after 1, 7 and 21 days in the hydrogel membranes (Figure 7A). Confocal images showed hMSCs growing on all the membrane surfaces at days 1 and 7. The number of living cells was much higher at day 21 and cells appeared covering the hydrogel membranes with few dead cells. These results indicated that Ch, Ch/HA and Ch/HAMA hydrogel membranes can provide an amenable environment that supports hMSCs growth and confirmed the cell viability with no cytotoxic effects.

Proliferation of hMSCs cultured in Ch, Ch/HA and Ch/HAMA hydrogel membranes was evaluated with AlamarBlue^®®^ assay at 1, 5, 7, 14 and 21 days of cell culture (Figure 7B). Results demonstrated that cell proliferation increased from day 1 until 21 days in all the systems. No significant differences were found between Ch/HAMA and Ch membranes regarding cell proliferation at any time. For its part, Ch/HA demonstrated a significantly enhanced cell proliferation in comparison to Ch at 5, 7, 14 and 21 days, and in comparison to Ch/HAMA system at 7, 14 and 21 days. This result may be due to the supportive microenvironment of semi-IPNs system for cell attachment and proliferation coming from entangled HA and a higher content of G_1_Phy in the semi-IPN system leading to a higher released concentration as it was observed in the G_1_Phy release profile (Figure 5), which after being released at short times of incubation could be assimilated by hMSCs, exerting a positive effect on cell adhesion and proliferation as it has been previously observed for similar polymeric systems [25,53]. Correira et al. [46] demonstrated that the biological performance of polysaccharides based systems was affected by their physicochemical factors, which could be tuned by the incorporation of HA to Ch at different proportions. In fact, they found that the addition of HA to Ch scaffolds up to 5% improved both physicochemical and biological properties of Ch scaffolds [46]. Additionally, previous works of the authors demonstrated the benefits of the glycerylphytate crosslinker on cell adhesion and proliferation of 3D scaffolds [25]. In overall, the better biological performance of the semi-IPN system can be attributed on the one hand, to its physicochemical features regarding surface roughness, mechanical properties, approaching those of cartilage, and wettability. On the other hand, the enhancement of cell viability and proliferation of Ch/HA sample in comparison to Ch/HAMA system can derived from the presence of linear HA embedded in the semi-IPN and its higher ability to be interchanged with the medium respect to crosslinked HAMA, which is longer retained in the IPN membrane [36,46], along with the higher content of the bioactive G_1_Phy for this membrane. Thus, in our study, the semi-IPN highlights as the best candidate to mimic the native tissue ECM. Some authors have claimed the benefits of semi-IPN systems containing HA for tissue regeneration due to its similarities to ECM composition [27,54]. For example, Pescosolido et al. [54] combined HA with photocrosslinkable dextran to overcome instability problems of HA derived from its high hydrophilicity. The presence of the bioactive HA provided excellent biological properties to their systems. For its part, Skaalure et al. [27] developed a semi-IPN consisting on poly(ethylene glycol) and entrapped HA, whose incorporation clearly led to an enhanced cell adhesion and proliferation.

## 4. Conclusions

Semi-IPN and IPN systems based on HA and Ch crosslinked with G_1_Phy were developed as biomimetic and degradable membranes with potential application in TE. Significant differences between semi-IPNs and IPNs were observed in terms of surface topography, mechanical, swelling and degradability properties. IPNs demonstrated to enhance HA retention, as well as mechanical properties of the polymeric network thanks to covalent crosslinking mediated by UV-light irradiation. Dual crosslinking processes of IPNs, consisting of ionic crosslinking of Ch and photopolymerization of HAMA, provided membranes with long-term stability and increased swelling. Moreover, the IPN framework led to flatter surfaces in comparison to semi-IPN. All the studied systems demonstrated high biocompatibility, supporting hMSCs adhesion and proliferation on their surfaces. However, the semi-IPN significantly increased cell proliferation over time respect to IPN, arising as the best candidate of the studied systems. This behavior could be due to the surface features of the semi-IPN (i.e., hydrophilic nature, granular topography and mechanical properties mimicking those of native cartilage), the higher content of the bioactive crosslinker and the entangled HA what seems to be key properties to favor hMSCs performance. These finding suggest that Ch/HA semi-IPNs ionically crosslinked with G_1_Phy have potential to be proposed as an effective promoter system of tissue repair. Further studies will be carried out to evaluate both in vitro and in vivo differentiation abilities of hMSCs seeded on these biomimetic ECM membranes and their potential application for guided bone regeneration.

## Figures and Tables

**Table 1:** Theoretical (Theo) and experimental (Exp) elemental compositions, crosslinker content, and yield percentage for Ch, Ch/HA and Ch/HAMA membranes.

Membrane Sample	C ^a^		H ^a^		N ^a^		C/N ^a^		G_1_Phy Content (%) ^b,c^	Yield (%)
	Theo	Exp	Theo	Exp	Theo	Exp	Theo	Exp		
Ch	44.9	43.03 ± 0.33	6.80	6.86 ± 0.01	8.60	8.24 ± 0.10	5.2	5.22 ± 0.02	5.4 ± 0.1	95.3 ± 1.1
Ch/HA	44.7	42.72 ± 0.11	6.53	6.77 ± 0.09	7.36	8.12 ± 0.11	6.1	5.25 ± 0.07	4.9 ± 0.5	75.0 ± 0.6
Ch/HAMA	44.7	42.11 ± 0.18	6.53	6.58 ± 0.15	7.36	7.21 ± 0.19	6.1	5.83 ± 0.15	2.5 ± 0.4	93.5 ± 2.5

^a^ Determined by elemental analysis. ^b^ Determined by ICP. ^c^ Gram of G_1_Phy per gram of membrane × 100.

## Supplementary Materials

The following are available online at https://www.mdpi.com/2073-4360/12/11/2661/s1, Figure S1: (A) ^1^H-NMR spectrum of HAMA with 4.5% methacrylation degree in D_2_O, and (B) ATR-FTIR spectra of HA and HAMA, Figure S2: ATR-FTIR spectra of Ch, Ch/HA, and Ch/HAMA membranes.
