# Supplementary material for: Evaluation of Glycerylphytate Crosslinked Semi- and Interpenetrated Polymer Membranes of Hyaluronic Acid and Chitosan for Tissue Engineering
Source: Polymers (Basel). 2020 Nov 11;12(11):2661. doi: 10.3390/polym12112661 (PMC7697555; doi:10.3390/polym12112661)
Supplement: Supplementary file 1 [file polymers-12-02661-s001.pdf]

Evaluation of glycerylphytate crosslinked semi- and interpenetrated polymer membranes of hyaluronic acid and chitosan for tissue engineering

Ana Mora-Boza [1,2,‡], Elena López-Ruiz [3,4,5,6,‡], María Luisa López-Donaire [1,2,*], Gema Jiménez [3,4,5,6], María Rosa Aguilar [1,2], Juan Antonio Marchal [3,4,6,7], José Luis Pedraz [2,8], Blanca Vázquez-Lasa [1,2,*], Julio San Román [1,2], Patricia Gálvez-Martín [9]

[1] Institute of Polymer Science and Technology, ICTP-CSIC. C/ Juan de la Cierva 3, 28006 Madrid, Spain.
[2] CIBER-BBN. Health Institute Carlos III, C/ Monforte de Lemos 3-5, Pabellón 11, 28029 Madrid, Spain.
[3] Biopathology and Regenerative Medicine Institute (IBIMER), Centre for Biomedical Research, University of Granada, Granada E-18100, Spain.
[4] Instituto de Investigación Biosanitaria de Granada (ibs.GRANADA), University Hospitals of Granada University of Granada, Granada E-18071, Spain.
[5] Department of Health Sciences, University of Jaén, 23071 Jaén, Spain.
[6] Excellence Research Unit "Modeling Nature" (MNat), University of Granada, Granada E-18016, Spain
[7] Department of Human Anatomy and Embryology, Faculty of Medicine, University of Granada, Granada E-18016, Spain.
[8] NanoBioCel Group, Laboratory of Pharmaceutics, University of the Basque Country (UPV/EHU), School of Pharmacy, Paseo de la Universidad 7, Vitoria- Gasteiz 01006, Spain.
[9] R&D Human Health, Bioibérica S.A.U., Barcelona, Spain.

Supplementary information

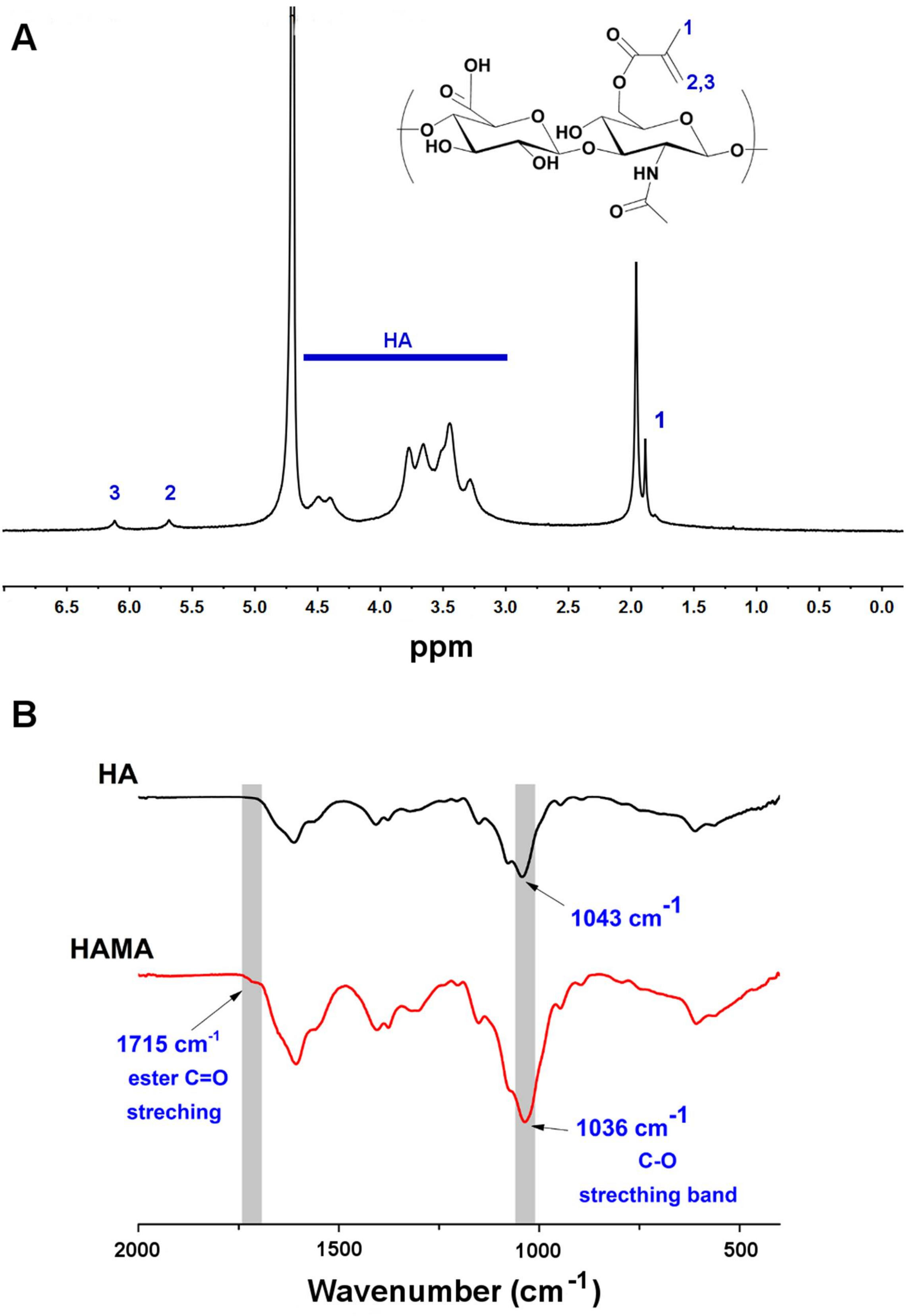

Figure. S1. (**A**) ^{1}H-NMR spectrum of HAMA with 4.5% methacrylation degree

in D_2O, and (**B**) ATR-FTIR spectra of HA and HAMA.

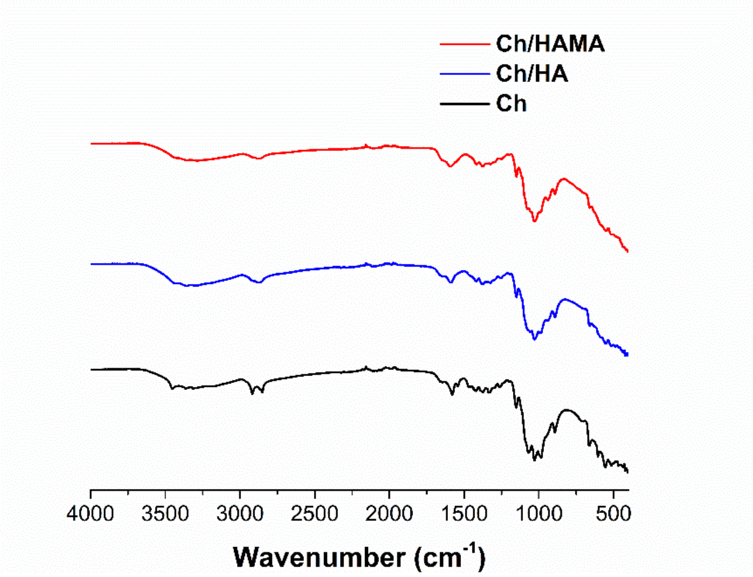

Figure. S2. ATR-FTIR spectra of Ch, Ch/HA, and Ch/HAMA membranes
